# Monitoring Coronavirus Disease 2019: A Review of Available Diagnostic Tools

**DOI:** 10.3389/fpubh.2021.672215

**Published:** 2021-06-07

**Authors:** Shanshan Liu, Qiuyue Li, Xuntao Chu, Minxia Zeng, Mingbin Liu, Xiaomeng He, Heng Zou, Jianghua Zheng, Christopher Corpe, Xiaoyan Zhang, Jianqing Xu, Jin Wang

**Affiliations:** ^1^Shanghai Public Health Clinical Center, Fudan University, Shanghai, China; ^2^Zhuhai Livzon Diagnostics Inc., Guangdong, China; ^3^School of Pharmacy, Gannan Medical University, Jiangxi, China; ^4^Department of Laboratory Medicine, Zhoupu Hospital Affiliated to Shanghai University of Medicine and Health Sciences, Shanghai, China; ^5^Nutritional Science Department, King's College London, London, United Kingdom

**Keywords:** COVID-19, SARS-CoV-2, rRT-PCR, immunoassay, coronavirus

## Abstract

Coronavirus disease 2019 (COVID-19) pneumonia is caused by the virus severe acute respiratory syndrome coronavirus 2 (SARS-CoV-2) and has rapidly become a global public health concern. As the new type of *betacoronavirus*, SARS-CoV-2 can spread across species and between populations and has a greater risk of transmission than other coronaviruses. To control the spread of SARS-CoV-2, it is vital to have a rapid and effective means of diagnosing asymptomatic SARS-CoV-2-positive individuals and patients with COVID-19, an early isolation protocol for infected individuals, and effective treatments for patients with COVID-19 pneumonia. In this review, we will summarize the novel diagnostic tools that are currently available for coronavirus, including imaging examinations and laboratory medicine by next-generation sequencing (NGS), real-time reverse transcriptase–polymerase chain reaction (rRT-PCR) analysis, immunoassay for COVID-19, cytokine and T cell immunoassays, biochemistry and microbiology laboratory parameters in the blood of the patients with COVID-19, and a field-effect transistor-based biosensor of COVID-19. Specifically, we will discuss the effective detection rate and assay time for the rRT-PCR analysis of SARS-CoV-2 and the sensitivity and specificity of different antibody detection methods, such as colloidal gold and ELISA using specimen sources obtained from the respiratory tract, peripheral serum or plasma, and other bodily fluids. Such diagnostics will help scientists and clinicians develop appropriate strategies to combat COVID-19.

## Introduction

Coronavirus (CoV) infections in humans primarily involve the upper respiratory and gastrointestinal tracts. Infections can result in a mild self-limiting disease similar to influenza or can become more severe life-threatening bronchitis and pneumonia with kidney involvement (1). The first human coronavirus (HCoV) was isolated from the mucus of a patient with influenza in 1965 and was known as B814 (2). Coronaviruses are enveloped viruses with a single-stranded, positive-sense RNA genome and have the largest known RNA virus genome of ~26–32 kb (3). Open reading frame 1a (ORF1a) mainly encodes non-structural proteins, such as enzymes related to viral replication and transcription, and ~1/4 of the genes at the 3′ end mainly encode surface spike (S) protein, membrane (M) protein, small envelope membrane (E) protein, and nucleocapsid (N) protein (4). Six coronaviruses are known to cause human diseases, including two members of the genus *Alphacoronavirus* HCoV-229E and HCoV-OC43 and four members of the genus *Betacoronavirus* HCoV-NL63, HCoV-HKU1, severe acute respiratory syndrome coronavirus (SARS-CoV), and Middle East respiratory syndrome coronavirus (MERS-CoV) (5). MERS-CoV is the pathogen that led to the outbreak of severe respiratory diseases in the Middle East in 2012 (6), and SARS-CoV is the cause of SARS in Guangdong Province of China in 2002 and 2003 (7–9).

A novel *Betacoronavirus*, SARS-CoV-2, that causes coronavirus disease 2019 (COVID-19) was first identified amid an outbreak of respiratory illness in Wuhan and named by the World Health Organization (WHO) on February 11, 2020 (10). Chan et al. (11) analyzed a family cluster of six people who returned to their homes in Shenzhen with infections after traveling to Wuhan, China. The incubation periods, clinical manifestations, and laboratory and radiological information of these patients with COVID-19 and the possible infection route of SARS-CoV-2 have been analyzed (12). Most patients presented with fever, dry cough, dyspnea, and computed tomography (CT) chest scans that revealed bilateral ground-glass opacities (GGOs). However, the characteristics of SARS-CoV-2 infection have few similarities to those of SARS-CoV and MERS-CoV infections (13, 14). Along with MERS-CoV and SARS-CoV, as the seventh member of the coronavirus family that infects humans, SARS-CoV-2 is more closely related to bat-SL-CoVZC45 and bat-SL-CoVZXC21 (15). At the cellular level, angiotensin-converting enzyme II (ACE2) is the receptor used by SARS-CoV-2 (16). Similar to SARS-CoV infection, SARS-CoV-2 enters cells through the receptor ACE2 and activates its spike protein through TMPRSS2 (17, 18). However, SARS-CoV-2 infections may be combined with a superspreader event under certain circumstances and transmitted on a large scale (19); this poses a high risk at the population level and will cause disruptions to the global public health system and economic losses (19). Thus, better methods for the early detection of this novel coronavirus are urgently needed.

**Figure 1:**
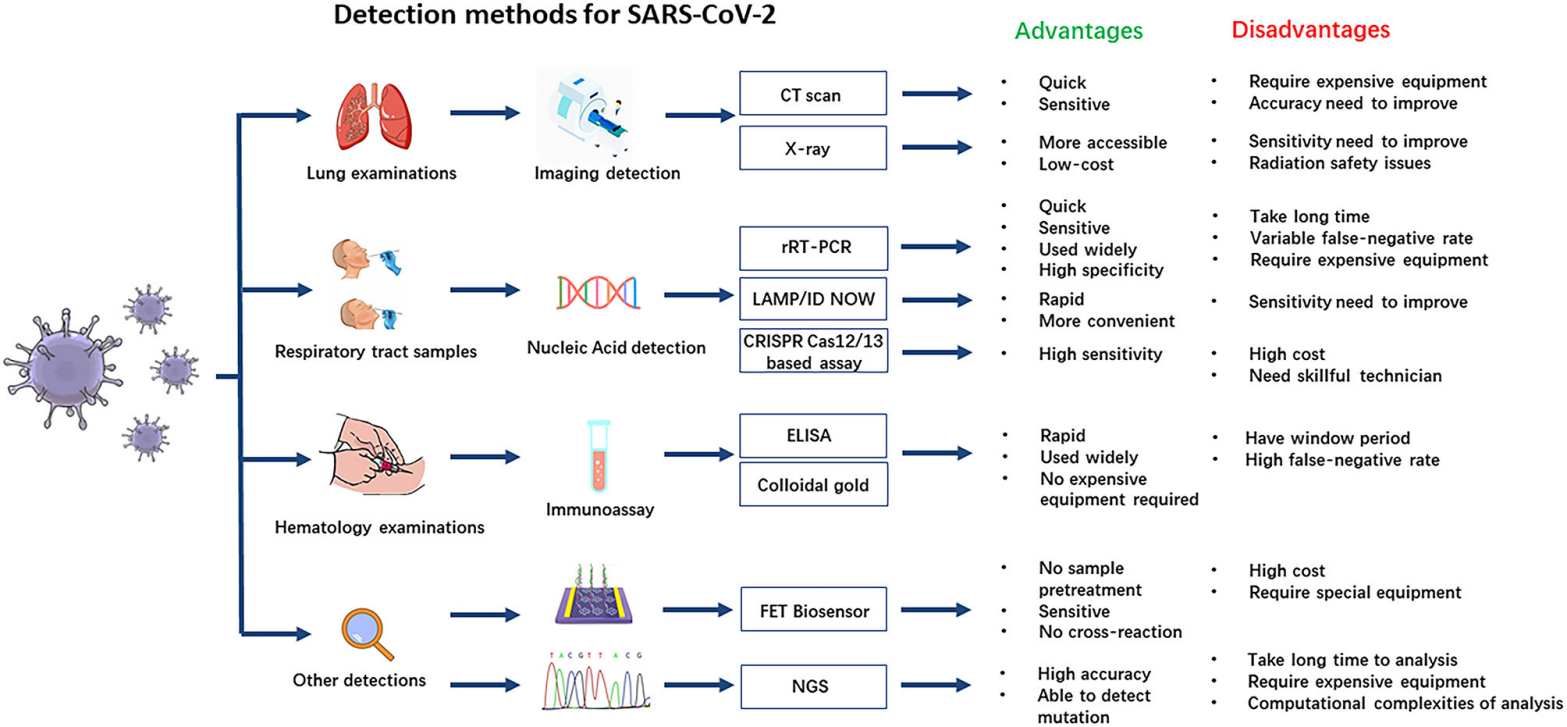
The detection methods for severe acute respiratory syndrome coronavirus 2 (SARS-CoV-2).

In clinical analysis laboratories around the world, real-time reverse transcriptase–polymerase chain reaction (rRT-PCR) is commonly used as an early detection method of the SARS-CoV-2 virus. rRT-PCR has several advantages, including reliability and high specificity, although analysis can take a long time, require expensive equipment, and produce quite variable false-negative rates (20). Immunoassays are a rapid bioanalytical method to detect an antigen–antibody that are widely used and take only about 20 min to give results, but immunoassays are not as specific as the tests recognizing viral RNA sequences, and during the early stages of infection may produce false-negative results (21). All the currently available diagnostic methods with their advantages and disadvantages are shown in Figure 1. Imaging examination is also a quick and sensitive diagnosis for COVID-19 in the early period, but it should be combined with other laboratory tests to improve its accuracy.

## Imaging Examinations for the Early Screening of Coronavirus Disease 2019

The imaging manifestations of COVID-19 on CT are similar to those of many other viral pneumonias, such as influenza, respiratory syncytial virus, and adenovirus infection, which make a differential diagnosis for COVID-19 difficult. For example, CT images in patients with SARS may show extensive disease and airspace consolidation (22), and MERS pneumonia appears on CT images as subpleural and basal airspace lesions with extensive GGOs and consolidation (23). Although the CT manifestations of COVID-19 have some similarities with those of MERS and SARS, CT is more sensitive for early stages of diseases (22), which still has a certain practicality when used for the early screening of COVID-19. The most common patterns of COVID-19 on thin-section CT images are pure GGOs that were defined as a hazy increase in lung attenuation with no obscuration of the underlying vessels and included reticular and/or interlobular thickened GGOs that are clearly distributed in the posterior and periphery of the lung (24). Among 21 patients with COVID-19 in China, GGOs were observed in 12 patients (57%), and consolidation was observed in six patients (29%) (25). COVID-19 is most likely to affect more than two lung lobes with bilateral involvement, with rapid evolution from focal, unilateral to diffuse, bilateral GGOs that progress to or coexist with consolidations within 1–3 weeks (26). CT is not specific for newborns, especially premature babies (27), and requires further scrutiny in case of diagnostic error. Although positive nucleic acid testing is the diagnostic gold standard, patients with fever and/or cough and prominent GGO lesions in the peripheral and posterior parts of the lungs on CT images, combined with normal or decreased white blood cells and a history of confirmed exposure, should be highly suspected of having COVID-19 (24). As a promising prognostic indicator for the clinical management of COVID-19, CT quantification of pneumonia lesions using artificial intelligence algorithms can predict the progression of serious diseases (28). A multicenter cohort study in 625 COVID-19 patients demonstrated that the consolidation in the upper lungs on initial CT was a risk factor associated with an adverse clinical outcome (29). The changes in CT images can help evaluate the treatment response of patients with COVID-19 (30). CT may also assist in the early detection of coronavirus cases. Chest radiography may also be considered to be a useful diagnostic tool for monitoring the rapid progression of lung abnormalities in COVID-19, particularly in the intensive care unit (ICU) (31). The sensitivity and specificity of chest CT were 85 and 50%, respectively, and sensitivity was significantly higher than that of X-ray (56%) (32). Compared to CT, chest radiography is cheaper and more accessible, which can minimize the risk of cross-infection (33). However, chest radiographs may provide a limited diagnosis because they may be normal in early or mild disease (34). Overall, imaging must be combined with other laboratory tests to establish the cause of the pneumonia observed by the CT scan. However, it still has certain limitations in terms of early diagnosis and differential diagnosis or correct diagnosis, and it cannot replace pathogen detection.

## Next-Generation Sequencing of Severe Acute Respiratory Syndrome Coronavirus 2

In the early stages of the pandemic, the first method used to detect the pathogen was next-generation sequencing (NGS) technology (35). This technique produces genome-wide coverage in a single experiment and measures each nucleotide position repeatedly (4, 36). Analyses of MERS-CoV genetics have been performed using capture-based NGS approach or deep genome sequencing for complete genome analysis (37, 38). Considering the high genetic diversity of bat coronavirus (39), NGS can ensure unbiased sequencing and quickly determine the nucleic acid sequence of this novel coronavirus. *De novo* SARS-CoV-2 genome sequences of clinical specimens (bronchoalveolar lavage fluid) and human airway epithelial virus isolates were obtained by Illumina and Nanopore sequencing (35). The viral genome sequence was released for immediate public health support *via* the community online resource virological.org on January 10, 2020 (Wuhan-Hu-1, GenBank accession number MN908947) (40), followed by four other genomes that were deposited on January 12 in the viral sequence database curated by the Global Initiative on Sharing All Influenza Data (GISAID) (41). However, unbiased NGS is an expensive method, and because of the high background level of non-viral sequences present in field monitoring samples, it is easy to miss low-abundance coronavirus sequences (38). With the emergence of the pandemic, a variety of COVID-19 detection kits have been rapidly developed worldwide, and as a result of the discovery of specific nucleic acid detection technology, the diagnostic value of NGS has decreased because of its high cost (42). In addition, the limitations of NGS include the long sample-to-result turnaround time (>2 days), limitations in the knowledge of how to interpret novel or rare mutations, and limited ability to detect structural gene variation and copy number variation (43).

## Real-Time Reverse Transcriptase–Polymerase Chain Reaction Analysis

rRT-PCR is more sensitive than conventional RT-PCR and has become the method of choice for the diagnosis of human coronaviruses (44). Sensitive rRT-PCR assays are essential for the rapid diagnosis of SARS-CoV in the early stages of the disease; multiple rRT-PCR assays have been developed to detect all four respiratory tract HCoVs that can be further adapted for the new CoV (45). SARS-CoV can be quickly detected by rRT-PCR analysis, which is based on multiple primers and probe sets located in different regions of the SARS-CoV genome and can distinguish SARS-CoV from other human and animal coronaviruses with a potential detection limit of <10 genomic copies per reaction. Clinical rRT-PCR testing has been shown to be suitable for the detection of SARS-CoV in clinical specimens and is valuable for the diagnosis of SARS-CoV infection. However, the effectiveness of rRT-PCR for the detection of SARS-CoV in clinical specimens is greatly affected by the number, type, and timing of specimen collection (46). False-negative results may also be caused by mutations in the primer and probe target regions in the SARS-CoV genome. rRT-PCR also remains the gold standard to confirm MERS-CoV. Upstream of the E gene (upE), ORF1a, ORF1b, and N genes are common targets for RT-PCR analysis of MERS-CoV (Table 1), while RNA-dependent RNA polymerase (RdRp), ORF1b, and N genes are targets for sequencing. The TaqMan probe-based one-step PCR assays allow for the rapid and sensitive internal diagnostic detection of MERS-CoV by detecting upE and ORF1b. In traditional RT-PCRs, step 1 is reverse transcription (RT) of RNA into cDNA, and step 2 is real-time PCR. The TaqMan probe-based one-step PCR kits use synthetic DNA templates and include the reverse transcriptase and DNA polymerase, which are premixed in a single reaction. The one-step method is reliable, specific, and reproducible (52). Compared to traditional RT-PCR, the one-step method combines two steps into one step, thus increasing the lower limits of sensitivity of the measurement; for example, <10 and ≤ 50 copies of RNA template per reaction are required for upE and ORF1b in MERS-CoV, respectively (52). ORF1ab/N gene nucleic acid assays are currently used for the detection of SARS-CoV-2. The expected amplicon sizes of the ORF1b and N gene assays for SARS-CoV-2 are 132 and 110 bp, respectively (53).

**Table 1:** Molecular detection primers and probes used for the real-time quantitative RT-PCR detection of coronaviruses.

**Coronavirus**	**Target**	**Forward primer (F)**	**Reverse primer (R)**	**Fluorescent probe (P)**	**Quencher probe (Q)**	**References**
SARS-CoV-2	ORF1ab	CCCTGTGGGTTTTACACTTAA	ACGATTGTGCATCAGCTGA	5'-FAM-CCGTCTGCGGTATGTGGAAAGGTTATGG-BHQ1-3'	-	(47)
	N genes	GGGGAACTTCTCCTGCTAGAAT	CAGACATTTTGCTCTCAAGCTG	5'-FAM-TTGCTGCTGCTTGACAGATT-TAMRA-3'	-	(47)
SARS-CoV	-	ACGGCTCTTCAGGAGTTGCTAA	TTCGTACTCACTTTC TTGTGCTTACA	5'-FAM-TGGATCCAATTTATGATGAGCCGACGA-3'	GCTCATCATAAATTGGATCCA-Dabcyl	(48)
	ORF1b	CAGAACGCTGTAGCTTCAAAAATCT	TCAGAACCCTGTGATGAATCAACAG	5'-FAM-TCTGCGTAGGCAATCC-NFQ-3'	-	(49)
	N genes	ACCAGAATGGAGGACGCAATG	GCTGTGAACCAAGACGCAGTATTAT	5'-FAM-ACCCCAAGGTTTACCC-NFQ-3'	-	(49)
MERS-CoV	S genes	TCGATCTTACCTACGAGATGTTGT	CAGCACAGTATGAAGAAGACGC	5'-FAM-CCGTGGTACATTTGGCTTGGTTTCA-TAMRA-3'	-	(50)
	ORF1a	CCACTACTCCCATTTCGTCAG	CAGTATGTGTAGTGCGCATATAAGCA	5'-FAM-TTGCAAATTGGCTTGCCCCCACT-TAMRA-3'	-	(51)
	upE	GCAACGCGCGATTCAGTT	GCCTCTACACGGGACCCATA	5'-FAM-CTCTTCACATAATCGCCCCGAGCTCG-TAMRA-3'	-	(51)

*FAM, 6-carboxyfluorescein; NFQ, nonfluorescent quencher; TAMRA, tetramethylrhodamine; SARS-CoV, severe acute respiratory syndrome coronavirus; MERS-CoV, Middle East respiratory syndrome coronavirus*.

rRT-PCR analysis of SARS-CoV-2 in the respiratory tract, urine, stool, and blood specimens has been developed by several companies internationally. Since the RNA is susceptible to hydrolysis, cold chain transportation must be used during sample delivery. Specimens that can be delivered promptly to the laboratory can be stored and shipped at 2–8°C (54). When there is likely to be a delay in specimens reaching the laboratory, specimens may be frozen to −20°C or ideally −80°C and shipped on dry ice if further delays are expected (54). After freezing and thawing more than four times, specimens will be damaged. A positive test for SARS-CoV-2 by rRT-PCR or gene sequencing revealing high homology to the SARS-CoV-2 gene can be used as a diagnostic criterion for suspected clinical cases (47). In clinical samples that have tested positive, the N gene assay is ~10 times more sensitive than the ORF1b gene assay in detecting positive clinical specimens (53). In the process of infection, subgenomic mRNA is necessary for virus replication. It contains multiple translation initiation sites and can encode structural proteins of the virus (55). It is possible that these clinical samples contain infected cells expressing subgenomic mRNA (56), resulting in more N gene copies in the samples. As these samples can only be qualitatively tested by these assays at the test site, the exact copy number in these samples cannot be determined (53). To confirm that a case is positive in the laboratory, the cycle threshold value in the rRT-PCR is the basis for judgment (a cycle threshold value ≤ 37 cycles is confirmed as positive in most labs) (57). According to the SARS-CoV-2 isolate Wuhan-Hu-1 (GenBank: MN908947.3), the conserved and specific regions are ORF1a (266-13468 nt), ORF1b (13469-21555 nt), and the N gene (28274-29533 nt) (58). The molecular detection primers and probes used for the rRT-PCR detection of SARS-CoV-2, SARS-CoV, and MERS-CoV are shown in Table 1. For SARS-CoV-2, nucleic acid extraction is performed by the one-step method or the use of magnetic beads. The one-step method requires no RNA extraction, and viral lysis, reverse transcription, amplification, and detection are achieved in a single-tube homogeneous reaction (59). The magnetic bead method utilizes nanotechnology to prepare superparamagnetic silicon oxide nanomagnetic beads, which can specifically identify and efficiently bind to nucleic acid molecules at the microscopic interface for RNA extraction (60). In Table 2, information is collected on several biotechnology companies undertaking the molecular diagnosis of COVID-19, including specimen source, methods of nucleic acid extraction, gene targeting, detection time, negative/positive percent agreement (NPA/PPA), and detection rate (DR). The analysis can be performed in as fast as 30 min with kits from Zhejiang Orient Gene Biotech Co., Ltd., Coyote Bioscience Co., Ltd. (57), and Hunan SANSURE BIOTECH INC. (57, 66). There are four companies (Cepheid, Diasorin Molecular, PerkinElmer, and Roche Diagnostics) on the list of SARS-CoV-2 diagnostic test kits by rRT-PCR analysis, which are updated on the Global Fund (GF) resources website based on eligibility criteria of WHO and US Food and Drug Administration (FDA) and Emergency Use Authorization (EUA) (69) and are shown in Table 2.

**Table 2:** Detection of SARS-CoV-2 nucleic acid by molecular technology methods.

**Company**	**Assay (nucleic acid extraction)**	**Amplification method**	**Specimen source**	**Target**	**Detection time (min)**	**PPA (%) (no.)**	**NPA (%) (no.)**	**DR (%)**	**Eligibility**	**References**
Applied Bio-Tech	Magnetic beads	Standard PCR	Respiratory tract	ORF1ab/N/E	90	100.0 (-)	100.0 (-)	100.0	NMPA/WHO	(57)
Bioperfectus Technologies	Magnetic beads	Standard PCR	Respiratory tract/Stool	ORF1ab/N/E	90	97.2 (970)	100.0 (970)	100.0	NMPA/WHO/US FDA EUA /TGA	(57, 61)
DAAN Gene	Magnetic beads	Standard PCR	Respiratory tract/Serum/Urine/Stool	ORF1ab/N	110	100.0 (-)	100.0 (-)	83.3	NMPA/WHO/TGA	(57, 61)
Zybio	Magnetic beads	Standard PCR	Respiratory tract/Serum/Urine/Stool	ORF1ab/N	60	100.0 (-)	100.0 (-)	100.0	NMPA	(57)
BGI Genomics	Magnetic beads	Standard PCR	Respiratory tract/Serum/Urine/Stool	ORF1ab	-	88.1 (126)	99.6 (258)	97.0	NMPA/US FDA EUA	(54)
Maccura Biotechnology	Magnetic beads	Standard PCR	Oropharyngeal swabs/nasopharyngeal swabs/nasal swabs	ORF1ab/N/E	-	100.0 (20)	96.7 (30)	-	US FDA EUA	(54)
Thermo Fisher	Magnetic beads	Standard PCR	Respiratory tract/Serum/Urine/Stool	ORF1ab/N/S	60	100.0 (-)	100.0 (-)	100.0	US FDA EUA	(54)
ZJ Bio-Tech	Magnetic beads	Standard PCR	Respiratory tract/Stool	ORF1ab/N/E	120	100.0 (252)	100.0 (252)	100.0	NMPA/WHO	(62)
Coyote Bioscience	One-step	Standard PCR	Respiratory tract	ORF1ab/N	30	-	-	66.6	NMPA/TGA	(57)
Easydiagnosis Biomedicine	One-step	Standard PCR	Respiratory tract	ORF1ab/N	75	95.9 (750)	94.1 (750)	94.8	NMPA/TGA	(63)
Orient Gene Biotech	One-step	Standard PCR	Respiratory tract	ORF1ab/N	30	-	-	97.0	NMPA	(64)
Promega	One-step		Respiratory tract	N	-	100.0 (13)	100.0 (104)	100.0	-	(65)
SANSURE Bio-Tech	One-step/Magnetic beads	Standard PCR	Respiratory tract/Serum/Urine/Stool	ORF1ab/N	30	100.0 (-)	100.0 (-)	100.0	NMPA/US FDA EUA/TGA	(57, 66)
Mammoth Biosciences	CRISPR-based DETECTR		Oropharyngeal swabs/Nasopharyngeal swabs	N/E	40	95.0 (30)	100.0 (30)	-	US FDA EUA	(67)
-	Cas13-based SHERLOCK	Isothermal	Nasopharyngeal and throat swab	ORF1ab/N/S	-	100.0 (154)	87.95 (154)	-	-	(68)
Biofire	-	Standard PCR	Nasopharyngeal Swab	ORF1ab/ORF8	-	100.0 (33)	100.0 (66)	100.0	US FDA EUA	(54)
Rutgers Clinical Genomics Laboratory	-	Standard PCR	Saliva	ORF1ab/N/S	-	100.0 (30)	100.0 (30)	-	-	(54)
Abbott ID Now	-	Isothermal	Nasal, nasopharyngeal and throat swabs	RdRp	13	94.0 (96)	100.0 (30)	-	US FDA EUA/TGA	(54)
Cepheid	-	Standard PCR	Nasopharyngeal Swab	N_2_/ E	30	97.9 (240)	100.0 (240)	-	WHO/US FDA EUA/TGA	(54)
Diasorin Molecular	-	Standard PCR	Nasopharyngeal swabs/nasal swabs/bronchoalveolar lavage	ORF1ab/S	-	100.0 (-)	100.0 (-)	100.0	WHO/US FDA EUA/TGA	(54)
PerkinElmer	-	Standard PCR	Oropharyngeal swabs/Nasopharyngeal swabs/Anterior nasal swabs	ORF1ab/N	-	100.0 (-)	100.0 (-)	95.0–100.0	WHO/US FDA EUA	(54)
Roche Diagnostics	-	Standard PCR	Nasopharyngeal and nasal swabs	ORF1ab	20	100.0 (-)	100.0 (-)	-	WHO/US FDA EUA	(54)

*Respiratory tract samples include throat swabs, sputum, and alveolar lavage fluid; one-step means no RNA extraction; NO. is number of samples tested; NPA, negative percent agreement; PPA, positive percent agreement; DR, detection rate; “-” is missing data; NMPA, National Medical Products Administration of China; US FDA EUA, Food and Drug Administration (FDA) and Emergency Use Authorization (EUA) of the United State; TGA, Therapeutic Goods Administration; SARS-CoV, severe acute respiratory syndrome coronavirus*.

Compared with conventional RT-PCR, the loop-mediated isothermal amplification (LAMP) is 100-fold-greater sensitivity for detection of SARS-CoV, with a detection limit of 0.01 PFU (70). By doing the LAMP assay, the virus is quickly extracted and amplified at constant temperature without the expensive reagents and equipment (71), but LAMP may be of lower sensitivity or comparable to SARS-CoV-2 detection by RT-PCR. Rödel et al. (72) demonstrated that the isolated RNA variplex RT-LAMP for SARS-CoV-2 had a sensitivity of 75% compared to LightMix E gene RT-PCR. On the other hand, the ID NOW COVID-19 assay performed on the ID NOW Instrument is a rapid (5–13 min) *in vitro* molecular diagnostic test, which utilizes isothermal nucleic acid amplification technology and amplification of the unique region of RdRP segment and nicking and extension amplification reaction (NEAC) where each primer consists of binding region and nicking enzyme recognition site. The newly synthesized short-sequence single-stranded chain is combined with the fluorescently labeled molecular probes to provide a real-time readout (54). The sensitivity and NPA of ID NOW can reach 87.58 and 96.99%, respectively. The saliva test has also demonstrated a high sensitivity and comparable performance to the current standard of nasopharyngeal and throat swabs and can significantly minimize the likelihood of exposing health care workers to SARS-CoV-2 while sampling (54, 73).

In addition to RT-PCR and isothermal amplification, clustered regularly interspaced short palindromic repeats (CRISPR)-based technologies have also been developed to detect the nucleic acids of SARS-CoV-2 in <40 min (67). This assay performs simultaneous reverse transcription and isothermal amplification, followed by Cas12 detection of SARS-CoV-2 sequences, after which cleavage of a reporter molecule confirms the detection of the virus. The kit provides a visual and faster method of detection, which showed 95% PPA and 100% NPA (67). It is a portable assay that enables point of care (POC) outside of the clinical diagnostic laboratory and replaces regular RT-PCR. Additionally, the specific high-sensitivity enzymatic reporter unlocking (SHERLOCK) assay using the enzyme Cas13a was developed for the detection of SARS-CoV-2, and the PPA of this assay is as high as 100% (68).

Specifically, some novel mutations of SARS-CoV-2 have already been identified as B.1.1.7, B.1.351, P.1, N501Y, and HV69-70del (74, 75). A one-step reverse transcription and real-time PCR (RT-qPCR) test is developed for screening Spike N501Y and HV69-70del mutations in 40 min. The specificity of this RT-qPCR assay relative to the sequencing-based technologies is 100% and can screen for SARS-CoV-2 efficiently (75).

## Immunoassay for Coronavirus Disease 2019

False-negative results are often obtained in the rRT-PCR analyses of coronavirus because nucleic acid assays can be affected by low virus copies or efficiency during amplification. For the detection of SARS-CoV antigens, a chemiluminescence enzyme-linked immunosorbent assay (CLEIA) can sensitively detect the target protein below 2 pg/ml at different stages of infection. The CLEIA shows no cross-reactivities to recombinant nucleocapsid (N) proteins of coronaviruses such as 229E, OC43, and NL63, and the specificity and sensitivity of this assay are both 100% (76). In addition, double-antibody sandwich enzyme-linked immunosorbent assays (ELISAs) based on specific monoclonal antibodies are used to detect the N proteins of HCoV-NL63 and HCoV-229E (77) and the SARS-CoV S protein of SARS-CoV (78). For the detection of MERS-CoV, an ELISA capture assay is developed to detect the N protein antigen of MERS-CoV virus in nasopharyngeal samples with high specificity (almost 100%) and sensitivity (<1 ng/ml) (79). Under an EUA, the Sofia 2 SARS Antigen Fluorescent Immunoassay (FIA) can be the practice of qualitative detection of N protein from SARS-CoV-2 in 15 min by immunofluorescence-based lateral flow technology for testing of patients suspected of COVID-19 (54).

Compared with rRT-PCR assays of upper respiratory tract samples, immunoassays are used to detect antibodies against SARS-CoV-2 from blood samples of patients suspected to have the active disease or to have had the disease in the past and thus are not used to detect the virus directly. The time window is also important for virus-specific antibody detection (80). Serology is the practice of detecting antibodies and can increase the sensitivity and specificity of SARS-CoV detection and is suitable for rapid laboratory diagnosis. SARS-CoV-2 proteins are used as the coated antigens to identify virus-specific IgM/IgG antibodies in blood samples of patients with COVID-19. The study showed that (81) IgM and IgG antibodies against SARS-CoV-2 were detected as early as the fourth day after the onset of symptoms and that IgG increased sharply by the 12th day. At 28 days, the seropositivity of IgG decreased, which were detected by ELISA kits from Livzon Diagnostics Inc. The sensitivity, specificity, and positive predictive value (PPV) of IgM antibodies were 77.3% (51/66), 100%, and 100%, respectively (81). The specificity and PPV of IgG–IgM combined detection assays are higher than those of individual IgG or IgM antibody assays (82). We also classify and describe the specimen sources, detection methods, detected antibodies, virus targets, detection times, and specificities and sensitivities of SARS-CoV-2 antibody detection kits according to the manufacturers' protocols (Table 3). The detection time of the colloidal gold method is short (15 min), and the virus targets are mainly S or N protein. The assay methods from Cellex using colloidal gold showed sensitivity and specificity to be more than 90%. The kit from Mount Sinai Laboratory also showed outstanding high sensitivity (92%) and specificity (100%). There are five companies (Advaite, Biocan Diagnostics, Biohit Healthcare, Biotest Biotech, and Laihe Biotech) on the list of diagnostic test kits using colloidal gold, which are updated on the GF resources website based on eligibility criteria of US FDA and EUA (69) and shown in Table 3. The ELISA from Beijing WANTAI BioPharm Co., Ltd., can simultaneously detect total antibodies (IgG/IgM/IgA). Moreover, the sensitivity and specificity of WANTAI BioPharm Co., Ltd., reach ~100% (83). The Chembio Dual Path Platform (DPP) COVID-19 IgM/IgG System is a single-use rapid immunochromatographic test for the qualitative detection of antibodies to SARS-CoV-2. The device employed Chembio's patented DPP technology that uses antibody capture to detect SARS-CoV-2 (54). If the sample contains SARS-CoV-2 antibodies, the conjugate binds to the antibodies captured in the test areas with more than 90% sensitivity and specificity. Rapid tests have great potential benefits for the prompt screening of COVID-19 infections. Thus, serological diagnostic approaches will aid in the diagnosis and treatment of novel coronavirus pneumonia and will also test the effectiveness of the vaccines and the selection of individuals who might act as plasma donors. However, rRT-PCR is widely adopted as the standard diagnostic method for SARS-CoV-2. Specifically, combining IgM and IgG detection methods with rRT-PCR assay results for the detection of the virus will greatly increase the accuracy of establishing the time of infection and predicting the progress of the disease (Table 4). Although the relationship between IgG levels in COVID-19 patients and protective immunity to SARS-CoV-2 virus has not been fully established (84), a positive result indicates an immune response to SARS-CoV-2 in COVID-19 patients. However, the patient is infected but has not yet produced IgG/IgM antibodies at detectable levels, resulting in false negatives (85). Also, cross-reactivity occurs when antibodies bind with an antigen, which is similar to the SARS-CoV-2 antigen, leading to a false-positive result. Thus, cross-reactivity is the biggest issue in the serological test.

**Table 3:** Immunoassays for COVID-19.

**Company**	**Assay (detection method)**	**Specimen source**	**SARS CoV-2 targets**	**Antibody detected**	**Time (min)**	**Sensitivity (%) (no.)**	**Specificity (%) (no.)**	**Eligibility**	**References**
Wandfo	Colloidal gold	Peripheral blood/ Serum/Plasma	S	IgG/IgM	15	86.4 (596)	99.6 (596)	NMPA	a
Innovita	Colloidal gold	Peripheral blood/Serum/Plasma	N	IgG/IgM	15	87.3 (126)	100.0 (126)	NMPA/US FDA EUA/TGA	b
Autobio Diagnostics	Colloidal gold	Serum/Plasma	S	IgG/IgM	15	88.2 (405)	99.0 (312)	NMPA	c
Cellex	Colloidal gold	Peripheral blood/Serum/Plasma	-	IgG/IgM	15	93.8 (128)	96.0 (250)	US FDA EUA/TGA	c
Mount Sinai Laboratory	ELISA	Serum/Plasma	S	IgG	-	92.0 (40)	100.0 (74)	-	c
Ortho Clinical Diagnostics	ELISA	Serum/Plasma	S	IgG/IgM	48	83.3 (36)	100.0 (400)	US FDA EUA	c
WANTAI BioPharm	ELISA	Serum/Plasma	S	IgG/IgM/IgA	120	100.0 (28)	98.0 (84)	NMPA/US FDA EUA/TGA	d
Chembio Diagnostic System	DPP	Peripheral blood/Serum/Plasma	N	IgG/IgM	15	93.5 (31)	90.2 (41)	-	c
Quidel	ELISA	Nasopharyngeal and nasal swab	N	-	15	80.0 (48)	100.0 (48)	US FDA EUA/TGA	c
Advaite	Colloidal gold	Peripheral blood	-	IgG	20	90.0 (30)	95.2 (104)	US FDA EUA	c
Biocan Diagnostics	Colloidal gold	Serum/Plasma	N/S	IgG/IgM	-	93.3(-)	96.2 (-)	US FDA EUA	c
Biohit Healthcare	Colloidal gold	Serum/Plasma	N	IgG/IgM	20	96.7 (30)	95.0 (80)	US FDA EUA	c
Biotest Biotech	Colloidal gold	Serum/Plasma	S	IgG/IgM	20	100.0 (30)	100.0 (30)	US FDA EUA	c
Laihe Biotech	Colloidal gold	Serum/Plasma	S	IgG/IgM	30	100.0 (30)	98.8 (80)	US FDA EUA	c

*NO. is the number of samples tested; NMPA, National Medical Products Administration of China; US FDA EUA, Food and Drug Administration (FDA) and Emergency Use Authorization (EUA) of the United State; TGA, Therapeutic Goods Administration; DDP, dual path platform; COVID-19, coronavirus disease 2019; SARS-CoV, severe acute respiratory syndrome coronavirus. (a) Guangzhou Wandfo Co., Ltd. (http://tech.gmw.cn/2020-02/24/content_33583856.htm); (b) INNOVITA Co., Ltd. (http://www.innovita.com.cn/html/cn/); (c) US Food & Drug administration (https://www.fda.gov/medical-devices/emergency-situations-medical-devices/emergency-use-authorizations); (d) Shenzhen Third People's Hospital in conjunction with Xiamen University and Beijing WANTAI BioPharm Co., Ltd. (https://www.medrxiv.org/content/10.1101/2020.04.09.20056325v1)*.

**Table 4:** Clinical significance of SARS-CoV-2 detection in COVID-19 patients.

**SARS-CoV-2nucleic acid assay**	**Immunoassay**		**Clinical significance**		
	**IgM**	**IgG**	Infection period^*^	**Immune response (Yes/No)**	**Other remarks**
+	+	+	Middle/Late	Yes	/
+	+	–	Early	Yes	/
+	–	+	Middle/Late	Yes	/
+	–	–	/	No	“Window period” for 2 weeks
–	+	+	/	Yes	Recovery/false-negative nucleic acid test
–	+	–	Early	Yes	http://www.baidu.com/link?url=0kXBuXMzX8-U029eB-yK4emFTye783LAu2tVaOpN8Am0q9DpIOpEzaFkTwUwFGcoK2rybVT1QQbEduUJEy4ar-mKBgjVCPwhOQ91QYDIjmReview for nucleic acid test
–	+/–	–	Early	No	Review after 1 week
–	–	+	/	Yes	Past exposure to SARS-CoV-2
–	–	–	/	/	Health/latent period (0–14 days)

* *Infection process is divided into early (0–7 days), middle (8–14 days), and late (after 14 days) periods for the onset of symptoms; “+” is positive and “-” is negative in detection analysis. COVID-19, coronavirus disease 2019; SARS-CoV, severe acute respiratory syndrome coronavirus*.

## Effect of Cytokines and T Cells on Coronavirus Disease 2019

SARS-CoV-2 can act as a factor for the development of a rapid autoimmune response that underlines COVID-19 outcomes. It is an important cause of acute respiratory distress syndrome and multiorgan illness that lasted for months in people with “long COVID” (86, 87). More than 10% of 987 patients with severe COVID-19 had antibodies that attacked and blocked the action of type 1 interferon, which could help to bolster the immune response against the virus (88) and played a key role in the pathophysiology of COVID-19 (89). After a novel coronavirus infection, pathogenic T cells are rapidly activated, and a large number of plasma cytokines and chemokines are produced, which causes a cytokine storm leading to severe immune damage to multiple organs (12, 90). Autoantibodies are more common in men than in women, and a poor T cell response negatively correlated with patients' age and was associated with worse disease outcome in male patients, which provides a possible explanation as to why COVID hits men harder (91). Cytokine storm refers to a phenomenon in which a large number of cytokines are rapidly released into bodily fluids after the patient is infected with microorganisms. Among T lymphocyte subpopulations in patients with COVID-19, both CD4^+^ and CD8^+^ T cell counts decreased, and the reduction in CD4^+^ T cells was more pronounced (42). However, in children, leukocyte counts and absolute lymphocyte counts were mostly normal (92). It was reported that interleukin (IL)-6 was elevated in more than half (52%) of patients with COVID-19 (90). In the early stage, initial plasma IL-1B, IL-1RA, IL-7, IL-8, IL-9, IL-10, basic fibroblast growth factor (FGF), granulocyte colony-stimulating factor (GCSF), granulocyte/macrophage colony-stimulating factor (GMCSF), interferon (IFN)γ, IFNγ-inducible protein (IP)10, monocyte chemotactic protein (MCP)1, macrophage inflammatory protein 1-alpha (MIP1A), MIP1B, platelet-derived growth factor (PDGF), tumor necrosis factor (TNF)α, and vascular endothelial growth factor (VEGF) concentrations were higher in all patients than those in healthy adults (12). The binding of COVID-19 to the Toll-like receptor (TLR) led to the release of pre-IL-1β, which was cleaved by caspase-1, followed by the activation of the inflammasome and the production of active mature IL-1β, which is a mediator of lung inflammation (62). Compared to those in non-ICU patients, IL-2, IL-7, IL-10, GCSF, IP10, MCP1, MIP1A, and TNFα levels were higher in ICU patients, suggesting that the cytokine storm was associated with disease severity (12). It was also found that the inflammatory factors IL-2R, IL-6, and C-reactive protein (CRP) were elevated in COVID-19 patients, and IL-2R and IL-6 had certain advantages in predicting the severity of the disease compared with traditional indicators (lymphocyte count and CRP) (93).

## Blood, Biochemistry, and Microbiology Laboratory Parameters of Coronavirus Disease 2019

In the early stage of COVID-19, the total number of white blood cells was generally reduced or normal, the lymphocyte count was decreased, and monocyte counts were increased or normal. If the absolute value of lymphocytes was <0.8 × 10^9^/L, the general recommendation was to review routine blood changes after 3 days. Leukopenia was observed in approximately 33.7% of the overall COVID-19 patient population. Among these patients, 82.1% had lymphopenia and 36.2% had thrombocytopenia. Moreover, lymphocytopenia, leukopenia, and thrombocytopenia were significant in severe cases of COVID-19 (94). The examination of peripheral blood cell morphology could show abnormal lymphocytes, and Dohle bodies could be found in the cytoplasm of some neutrophils (92).

In 99 cases of COVID-19 in Wuhan, most patients had common inflammation-like biochemical indicators on admission. Seventy-three patients were tested for CRP, of whom 63 (86%) patients had increased levels of CRP. Forty-three of 99 patients had differing degrees of liver damage, as shown by abnormal levels of alanine aminotransferase (ALT) or aspartate aminotransferase (AST). A large number of patients, ~98%, had decreased serum albumin levels. In addition, there was an increase in serum ferritin (FER) in 62 (63%) patients, an increase in erythrocyte sedimentation rate (ESR) in 84 (85%) patients, and an increase in the levels of blood glucose in 51 (52%) patients (90). In another report on COVID-19 (12), levels of lactate dehydrogenase (LDH) were elevated above 245 U/L in 29 of 40 (73%) patients, including 12 of 13 (92%) patients requiring ICU care and 17 of 27 (63%) of non-ICU patients. Of the 40 patients, five (12%) patients had levels of hypersensitive troponin I (hs-cTnI) above 28 pg/ml, which was due to virus-related cardiac injury, including 4 of 13 (31%) patients in the ICU. Procalcitonin (PCT) is a protein used to determine whether there is a bacterial infection in the lungs (95). On admission, the levels of PCT were in the normal range (PCT <0.1 ng/ml) for 27 (69%) of 39 patients. PCT levels were increased above 0.5 ng/ml in only three (8%) of the 39 patients. Among the 39 patients, there were four ICU patients who developed secondary infections. Three of the four patients with secondary infections had PCT levels >0.5 ng/ml. The arterial oxygen saturation (SpO_2_) and oxygen partial pressure decreased and carbon dioxide partial pressure increased in some severe cases of COVID-19, and those with metabolic acidosis occasionally had decreased pH (92).

COVID-19 is very different from other known viral pneumonia, such as influenza virus, adenovirus, respiratory syncytial virus, and mycoplasma pneumonia infections. Routinely detected influenza antigens are the A, B, and H7N subtypes (42). Due to the rapid detection method, the sampling of throat swabs can help to screen for influenza early in the clinical course, but the false-negative rate of the method is high (42). Therefore, the diagnosis of COVID-19 should be combined with nucleic acid, antibody, and antigen detection technology to improve the DR.

## Rapid Detection of Coronavirus Disease 2019 Severe Acute Respiratory Syndrome Coronavirus 2 Using a Field-Effect Transistor-Based Biosensor

A field-effect transistor (FET)-based biosensing device has been invented recently for detecting SARS-CoV-2 in clinical samples. FET-based biosensors are considered to be potentially useful in clinical diagnosis, POC testing, and on-site detection (96). The sensor is fabricated by coating graphene sheets of the FET with a specific antibody against SARS-CoV-2 spike protein. The sensor is able to detect the SARS-CoV-2 spike protein at concentrations of 1 fg/ml in phosphate-buffered saline and 100 fg/ml clinical transport medium, which makes it a highly sensitive immunoassay for COVID-19. Additionally, the biosensor can avoid cross-reaction with MERS-CoV antigen, indicating that the FET sensor is sensitive and specific enough for the SARS-CoV-2 spike protein. This method does not require sample pretreatment and labeling, and the biosensor does not cross-react with SARS-CoV and MERS-CoV antigens due to high specificity to SARS-CoV-2 spike protein by the selected antibody (96).

## Summary and Prospects for the Detection of Severe Acute Respiratory Syndrome Coronavirus 2 in Coronavirus Disease 2019

Coronavirus-related diseases have become an urgent global public health problem, and the detection of coronavirus is particularly important in the diagnosis of coronavirus diseases. RT-PCR has been widely used in diagnostic virology, and skilled diagnostic laboratories can rely on this powerful technique to internally establish new diagnostic assays during public health emergencies. In the face of a sudden outbreak, rapid and accurate detection and triage, with the isolation or treatment of suspected and confirmed cases, are the most powerful measures to prevent further spread of disease. Therefore, the SARS-CoV-2 nucleic acid assay is an important medical test for prevention, control, and medical treatment during an epidemic.

For suspected cases of COVID-19, rapid antigen and multiple PCR nucleic acid detection methods should be adopted as widely as possible. The detection of nucleic acids is the leading advance in the current clinical diagnostic technology for pathogenic microorganisms and is guiding the direction of lung infection diagnostic technology. Most commonly, the DR of this method is over 90%, and NPA and PPA are close to 100%, and this method is widely used in the clinical diagnosis and detection of various respiratory pathogens. However, with the widespread use of SARS-CoV-2 nucleic acid assays, an increasing number of problems are becoming apparent. The major issue is a high number of false-negative results in virus nucleic acid detection.

For rRT-PCR analysis, the results of the nucleic acid assay are affected by factors such as the disease development process, specimen collection, specimen preservation and transportation, nucleic acid extraction, amplification system, detection operating environment, and personnel operations. The concentration of SARS-CoV-2 in the alveolar lavage fluid of COVID-19 patients is the highest, but the procedure of collecting alveolar lavage fluid is complicated, it is only recommended for critically ill patients on ventilators. However, the most common and simplest sampling method remains a throat swab. Moreover, the correct handling of specimens during transportation is essential. Specimens for virus detection should be kept cold and stored at −70°C if the testing is to be delayed for a long time. Most of the (RNA) vaccines also require logistics for storage at very low temperatures. Previously, it was stated by Shanghai ZJ Bio-Tech Co., Ltd., that because their kit requires a storage environment of −20°C, only a few logistics companies with cold chain capabilities could supply the kit. Carelessness during transportation may affect the final test results of the assays. On the other hand, many sampling solutions cannot lyse the virus and cannot guarantee the stability of the viral nucleic acid, which is one of the factors that affects detection sensitivity.

Testing SARS-CoV-2-specific antibodies in patient blood is a good choice for rapid, simple, and highly sensitive diagnosis of COVID-19, which can also meet the urgent needs of a large number of patients. Hence, immunoassays are an excellent supplementary approach in clinical applications. Moreover, monoclonal antibodies have been developed against SARS-CoV-2 antigen proteins, which can target a single specific epitope and are highly specific compared to polyclonal antibodies. Overall, compared with polyclonal antibodies, monoclonal antibodies have been indicated to be efficient reagents in terms of specificity for clinical diagnostic tests, which is greatly valuable for clinical detection. Some of the antibodies do not react with the new emerging mutant variants of the virus. Therefore, the diagnosis of COVID-19 needs to be further improved to reduce the misdiagnosis rate and to adapt to new mutated versions of SARS-CoV-2. Therefore, the diagnosis of COVID-19 needs to be further improved to reduce the misdiagnosis rate. Multiple detection methods can be used together to improve the correct diagnosis rate.

With the increase in the prevalence of SARS-CoV-2 variants containing spike mutations, a one-step rRT-qPCR test needs be developed that will rapidly detect mutations with low cost. When the evolution of the pandemic causes the number of asymptomatic SARS-CoV-2-positive individuals and patients with COVID-19 to grow exponentially, fully automated immunoassays and PCRs, capable of executing thousands of tests per day, have gained importance. New technologies, such as the POC diagnosis device, provide a portable, fast, and low-cost assay system for the diagnosis of COVID-19 that can be used not only in the doctor's offices but also in homes, airports, and remote locations. The integration of smartphones with COVID-19 detection technologies should be considered a promising testing platform in the future. Additionally, the development of a novel assay for coronavirus pneumonia still has a long way to go, but it will greatly contribute to the clinical diagnosis of COVID-19.

## Author Contributions

JW conceived and designed the study. SL, MZ, XH, HZ, and ML collected the literature. SL drafted the paper. JW, CC, and QL revised the manuscript with input from all coauthors. JW, XZ, and JX provided funding support. XC and JZ were the principal investigators for the detection of SARS-CoV-2. All authors read, contributed to, and approved the final manuscript.

## Conflict of Interest

XC and MZ were employed by the company Zhuhai Livzon Diagnostics Inc. The remaining authors declare that the research was conducted in the absence of any commercial or financial relationships that could be construed as a potential conflict of interest.

## Glossary

ACE2, angiotensin-converting enzyme II; ALT, alanine aminotransferase; AST, aspartate aminotransferase; CLEIA, chemiluminescence enzyme-linked immunosorbent assay; CoV, coronavirus; COVID-19, coronavirus disease 2019; CRP, C-reactive protein; CT, computed tomography; DPP, dual path platform; DR, detection rate; ELISA, enzyme-linked immunosorbent assay; ESR, erythrocyte sedimentation rate; FET, field-effect transistor; FER, ferritin; GISAID, Global Initiative on Sharing All Influenza Data; GGOs, ground-glass opacities; HCoV, human coronavirus; hs-cTnI, hypersensitive troponin I; LAMP, loop-mediated isothermal amplification; LDH, lactate dehydrogenase; MERS-CoV, Middle East respiratory syndrome coronavirus; NGS, next-generation sequencing; NPA, negative percent agreement; N protein, nucleocapsid protein; ORF1ab, open reading frame 1ab; POC, point of care; PCT, procalcitonin; PPA positive percent agreement; PPV, positive predictive value; PGV, ground-glass opacity volume; RdRp, RNA-dependent RNA polymerase; rRT-PCR, real-time reverse transcription polymerase chain reaction; SARS-CoV-1, severe acute respiratory syndrome coronavirus 1; S protein, spike protein; SpO_2_, oxygen saturation; TLR, Toll-like receptor; upE, upstream of the E protein; VL, viral load; WHO, World Health Organization.
